# Impact of Middle-Aged Adults’ Recognition of Early Myocardial Infarction Symptoms and Training Experience on Cardiopulmonary Resuscitation Performance: A Cross-Sectional Study

**DOI:** 10.3390/ijerph22010054

**Published:** 2025-01-01

**Authors:** Dajung Ryu

**Affiliations:** Department of Nursing, Kyungmin University, Uijeongbu-si 11618, Republic of Korea; icurdj@naver.com; Tel.: +82-31-828-7479

**Keywords:** myocardial infarction, cardiac arrest, cardiopulmonary resuscitation, automated external defibrillators

## Abstract

Despite the increasing incidence of myocardial infarction among middle-aged adults, studies analyzing their recognition of early myocardial infarction symptoms and cardiopulmonary resuscitation training experiences are lacking. This study aimed to utilize data from the 2022 Korea Community Health Survey to assess the level of recognition of early myocardial infarction symptoms among middle-aged adults and identify factors affecting cardiopulmonary resuscitation performance based on training experiences. Secondary data analysis was conducted to assess 99,945 adults aged 40–64 years on their recognition of early myocardial infarction symptoms and the influence of cardiopulmonary resuscitation training experience on their performance ability. Data analysis was performed using multinomial logistic regression, followed by assessing the area under the curve and visualizing the receiver operating characteristic curve to evaluate the model’s performance. The recognition of early myocardial infarction symptoms improved; the cardiopulmonary resuscitation performance ability increased by 22%. The absence of training with mannequins and automated external defibrillators resulted in a 79% and 77% decrease in cardiopulmonary resuscitation performance ability, respectively. Enhancing the recognition of early myocardial infarction symptoms and providing hands-on cardiopulmonary resuscitation training is vital for improving cardiopulmonary resuscitation performance in middle-aged adults. Effective cardiopulmonary resuscitation training programs can facilitate the rapid identification of patients who have had a cardiac arrest, bolster emergency response capabilities, and enhance the overall social safety net.

## 1. Introduction

The three leading causes of human death globally are cardiovascular disease, stroke, and chronic obstructive pulmonary disease [1]. In 2021, cardiovascular diseases were the leading cause of mortality worldwide [1], with myocardial infarction (MI) responsible for most of the 17.9 million cardiovascular-related deaths reported in 2019 [2,3]. Historically, the incidence of MI was believed to increase with age due to severe myocardial damage [4]. However, chronic diseases, such as hypertension, stroke, and hyperlipidemia, have an earlier onset, making MI increasingly common among middle-aged adults. In the United States, the hospitalization rate of middle-aged adults for MI increased from 27% in 1999 to 32% in 2014, with the one-year post-cardiac arrest mortality rate reaching 10% [5]. Approximately 47% of patients with MI in China are middle-aged adults [3], and in South Korea, the incidence rate of cardiac arrest among middle-aged adults is as high as 74.5% [6,7,8].

Cardiac arrest is a complex condition where structural or functional impairments in the heart make normal blood circulation impossible, halting blood flow to the brain and vital organs [9]. Most out-of-hospital cardiac arrests occur in community settings and have high mortality rates, posing a significant public health burden [10]. The Korea Disease Control and Prevention Agency recommends that all citizens learn and perform cardiopulmonary resuscitation (CPR), with training provided to healthcare professionals and the public by the Korean Association of Cardiopulmonary Resuscitation. Factors affecting CPR performance, such as knowledge, confidence, teamwork, skills, and self-efficacy, have been studied, but most research is limited to in-hospital cardiac arrests or focuses on medical professionals [11,12,13].

The effective performance of CPR by the first witness of a cardiac arrest is the most crucial factor in increasing patient survival rates. Bystander CPR improves survival rates by 10% per minute [14]. Middle-aged adults are likely to encounter emergencies such as cardiac arrest at home and work, and anyone in the community can become a bystander, necessitating accurate CPR skills. Enhancing CPR performance capabilities in this population can significantly improve life-saving effectiveness and emergency response abilities. Additionally, as role models for younger generations, middle-aged adults can emphasize the importance of CPR knowledge and skills, contributing to improved emergency response capabilities across society. Thus, strengthening the CPR performance abilities of middle-aged adults provides critical benefits both individually and socially.

Despite the increasing incidence of MI among middle-aged adults, research analyzing their recognition of early MI symptoms and CPR training experiences is lacking. Some studies have examined the effects of external interventions, including telephone dispatcher-assisted CPR, on bystander CPR performance [15,16]; however, more research is needed on factors related to the bystander initiation of CPR in out-of-hospital cardiac arrest situations.

This study aimed to utilize data from the 2022 Korea Community Health Survey [17] to assess the level of recognition of early MI symptoms among middle-aged adults and identify factors affecting CPR performance based on CPR training experiences. The findings are intended to provide foundational data for developing CPR training programs for middle-aged adults.

## 2. Materials and Methods

### 2.1. Study Design

This is a cross-sectional study with secondary data analysis aimed at identifying factors affecting CPR performance ability among middle-aged adults, using data from the 2022 Korea Community Health Survey conducted by the Korea Disease Control and Prevention Agency.

### 2.2. Study Participants and Data Collection

The Community Health Survey data is public and recognized as national statistics (approval number 117075). Sample extraction was conducted using probability proportional to size systematic sampling based on resident registration population data. The survey participants were adults aged ≥ 19 years old living in sampled households. Data were collected through face-to-face interviews by trained investigators from August to October 2022. The total number of participants was 231,785, and this study focused on 99,945 middle-aged adults aged 40–64 years old who responded to all survey variables.

### 2.3. Measurements

#### 2.3.1. Recognition of the Early Symptoms of MI

The recognition of early MI symptoms was assessed using five items: “sudden pain or discomfort in the jaw, neck, or back”, “sudden chest pain or pressure or a squeezing feeling”, “sudden pain or discomfort in the arms or shoulders”, “sudden weakness, dizziness, nausea, or cold sweat”, and “sudden shortness of breath”. Responses to the five items were recorded as binary outcomes (yes or no). The individual item scores were summed to compute a total score ranging from 0 to 5, where higher scores indicated a greater level of recognition of the early MI symptoms.

#### 2.3.2. CPR Training Experience: Mannequin and Automated External Defibrillators (AED) Practice

CPR training experience refers to whether the participants had practiced CPR using mannequins and AEDs in the previous two years. CPR training refers to a structured, instructor-led program lasting at least 40 min, conducted at health centers, fire stations, educational institutions, military facilities, or through organizations such as the Korean Association of Cardiopulmonary Resuscitation. Mannequin practice refers to participants having practiced chest compressions on a mannequin, and AED practice refers to participants having practiced using an AED. The questions were “Have you had mannequin practice experience during CPR training in the past two years?” and “Have you had AED practice experience during CPR training in the past two years?” with binary response options (yes or no).

#### 2.3.3. CPR Performance Ability

CPR performance ability refers to the capability to perform CPR when witnessing a cardiac arrest. The question was “Can you perform CPR if you witness a cardiac arrest?” with response options “can perform accurately”, “can perform approximately”, and “cannot perform” rated on a three-point scale. The response “can perform accurately” encompassed the ability to properly perform chest compressions, rescue breaths, and use an AED, whereas the response “can perform approximately” indicated the ability to perform some parts of CPR.

### 2.4. Statistical Analysis

Data analysis was conducted using SPSS 25.0, Chicago, IL, USA. The data of middle-aged adults aged 40–64 years were extracted from the entire dataset. Descriptive statistics were used to analyze general characteristics and variable levels. As accurate CPR performance can enhance survival rates and improve outcomes for patients undergoing cardiac arrest [18], those who responded that they could perform CPR accurately were classified into the perfect CPR performance group, whereas those who responded that they could perform approximately or could not perform were classified into the comparison group. The differences between the two groups (perfect CPR performance and comparison groups) were analyzed using chi-square tests for categorical data, Cramer’s V for measuring the strength of the association between categorical variables, and *t*-tests for comparing the means of continuous variables between the two groups. Pearson correlation coefficients were used to identify correlations between analysis variables, and multinomial logistic regression analysis was conducted to identify factors affecting CPR performance ability. Given that CPR performance responses were categorized on a three-point ordinal scale, either ordinal logistic regression or multinomial logistic regression methods were suitable. Ordinal logistic regression follows the proportional odds model, assuming that independent variables have the same effect on each level of the dependent variable, thus requiring a test of parallel lines (TPL). In this study, the TPL significance probability was less than 0.05, leading to the use of multinomial logistic regression analysis. Sampling weights were applied to adjust for demographic imbalances to ensure the results were more representative of the general population. The model’s performance was evaluated by checking the area under the curve (AUC) and visualizing the receiver operating characteristic curve. A significance threshold of 0.001 was used for all statistical tests.

## 3. Results

### 3.1. General Characteristics of the Study Participants

This study included 99,945 participants, with females accounting for 53.2% of the sample. The average age was 53.11 years, with the majority (41.6%) of the participants being in their 50s. Household composition was distributed as follows: 52.6% were in two-generation households, 42.1% in one-generation households, and 5.3% in three-generation households. The highest educational level was college graduation or above, representing 43.5% of the participants. Economic activity participation was reported by 76.5% of respondents, and 70.7% resided in provinces. Additionally, 41.2% of the participants had no experience with CPR training, whereas 22.8% had received CPR training within the previous two years. When distinguishing between groups based on accurate CPR performance ability, all variables, except for household composition (χ^2^ = 1.06, *p* = 0.60), exhibited significant differences (Table 1).

### 3.2. Recognition of the Early Symptoms of MI

The average recognition score of early MI symptoms among all study participants was 3.82. Although 91.1% of the participants recognized chest pain as a symptom, 39.1% were unaware that pain in the arms and shoulders could also be an early MI symptom. When participants were stratified based on their CPR performance ability, the average score was significantly higher in the group with accurate CPR performance (4.11) than that in the group with inaccurate performance (3.81), with this difference being statistically significant (χ^2^ = −24.65, *p* < 0.001). The five symptoms, i.e., chest pain; shortness of breath; dizziness; pain in the jaw, neck, and back; and pain in the arms and shoulders, were identified in descending order of recognition levels (Table 2).

### 3.3. Correlation Between the Early Symptoms of MI

Among the five early symptoms of MI, a weak positive correlation was observed between chest pain and pain in the jaw, neck, and back (r = 0.38, *p* < 0.001); pain in the arms and shoulders and shortness of breath (r = 0.37, *p* < 0.001); and chest pain and pain in the arms and shoulders (r = 0.30, *p* < 0.001). Other variables exhibited moderate positive correlations (Table 3).

### 3.4. Factors Influencing CPR Performance Ability

Multinomial logistic regression analysis was conducted to identify the factors affecting CPR performance ability. The model’s fit was statistically significant with −2LL = 340.46, χ^2^ = 2136.62, *p* < 0.001, and the explanatory power was 11.0% (Nagelkerke R^2^ = 0.11). An increase of one point in MI early symptom recognition was associated with a 9.0% increase in ability to perform CPR approximately (odds ratio (OR) = 1.09, 95% confidence interval (CI) = 1.05–1.13) and a 22.0% increase in ability to perform CPR accurately (OR = 1.22, 95% CI = 1.18–1.26). In situations where approximate CPR performance was feasible, lacking mannequin training experience resulted in a 51.0% decrease in CPR performance ability (OR = 0.49, 95% CI = 0.43–0.56), and in situations where accurate CPR performance was feasible, it led to a 79.0% decrease (OR = 0.21, 95% CI = 0.18–0.25). Similarly, in situations where approximate CPR performance was feasible, lacking AED training experience led to a 46.0% decrease in CPR performance ability (OR = 0.54, 95% CI = 0.48–0.60), and in situations requiring accurate CPR performance, it resulted in a 77.0% decrease (OR = 0.23, 95% CI = 0.20–0.26) (Table 4).

**Figure 1:**
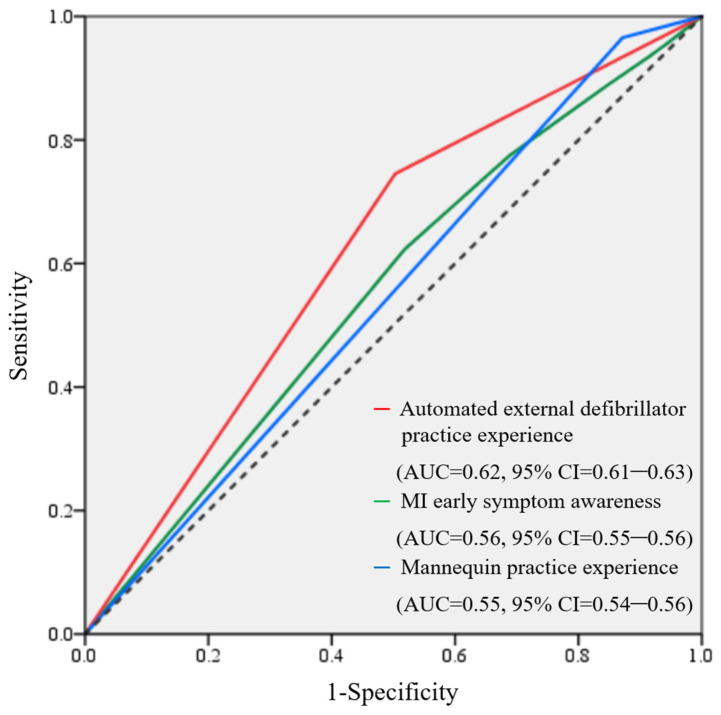
The receiver operating characteristic curves.

The AUC values were assessed to evaluate the model’s performance. The values for AED practice experience, MI early symptom recognition, and mannequin practice experience were 0.62 (95% CI = 0.61–0.63), 0.56 (95% CI = 0.55–0.56), and 0.55 (95% CI = 0.54–0.56), respectively, indicating that the model was appropriate as the AUC values were greater than 0.5 (Figure 1).

## 4. Discussion

With conditions leading to cardiac arrest becoming increasingly prevalent, including MI, hypertension, and stroke, enhancing the ability of the general public to perform CPR can significantly reduce mortality rates. The effective resuscitation of patients undergoing cardiac arrest involves a continuous sequence of these five links in the survival chain: (1) the recognition of cardiac arrest and calling for help, (2) bystander CPR, (3) defibrillation, (4) advanced life support, and (5) post-resuscitation care. This study was conducted to identify factors affecting the CPR performance ability of middle-aged adults, providing a basis for developing programs to strengthen the survival chain. Our main findings indicate that with each incremental increase in the recognition of MI early symptoms, CPR performance ability improved by approximately 9–22%. In contrast, the lack of experience with mannequin training resulted in a 51–79% reduction in CPR performance ability, and the absence of AED training experience led to a 46–77% decrease in performance ability.

Only 15.5% of the 99,945 middle-aged adults could perform CPR accurately. Although direct comparisons are challenging due to limited studies on middle-aged adults’ CPR performance, this figure is lower compared to those reported in Japan (40.2%), Singapore (24.3%), Malaysia (22.6%), and Thailand (15.8%) [19]. The immediate initiation of CPR by the first bystander is crucial for improving survival rates [14,20]; however, the general CPR performance rate among all age groups in Korea was only 29.2% in 2022 [21]. This suggests the need to address barriers to CPR training and participation, considering factors such as age, understanding, infrastructure, and educational information, and to promote tailored educational programs.

Over 60% of the study participants were aware of the five early symptoms of MI. However, those who could not perform CPR accurately had lower awareness rates for each symptom than those who could perform CPR accurately. Particularly, recognition rates for pain in the jaw, neck, and back, as well as pain in the arms and shoulders, as MI early symptoms were lower. This is consistent with the results of a systematic review of MI early symptom awareness among the public in 35 countries, which found that the recognition of chest pain was the highest, whereas the awareness of jaw, neck, and back pain was lower [22]. The knowledge of MI early symptoms may vary by age, race, and socioeconomic status [23], and one of the key goals of CPR education is to enable the public to quickly recognize and intervene in cardiac arrest situations to minimize delays and save lives, as incomplete awareness can lead to the delayed or missed initiation of CPR [24,25]. As the public may find it challenging to connect pain in the jaw, neck, back, arms, and shoulders with early MI symptoms, future CPR education programs should emphasize this knowledge to strengthen the first link in the survival chain. Furthermore, understanding the reasons for the lower recognition rates for certain symptoms could inform targeted interventions. For instance, tailored education strategies that focus on the importance of recognizing atypical symptoms, such as pain in the jaw and shoulders, could improve overall awareness and preparedness for MI.

The association between CPR training experience and CPR performance ability is well-established in the literature [24,26,27]. Moreover, practical training with equipment has been reported to markedly enhance CPR skills compared to lecture-based training without hands-on practice [28]. Trained individuals are highly likely to start CPR faster, report to emergency medical services, and use an AED three times more frequently [26]. Practical training experiences thus positively influence the first three stages of the survival chain, improving survival rates. However, CPR training coverage varies globally, ranging from 3–79%, and the proportion of the public receiving training within the past two years remains relatively low [27]. Regular opportunities for practical training should be provided, and efforts should be made to streamline training programs and enhance accessibility. For instance, Japan has incorporated CPR training into school curricula and driver’s license requirements [19], and Korea is producing and distributing educational videos to facilitate easy learning of CPR [21]. Nonetheless, training for AED use and the recognition of cardiac arrest situations are lacking. Therefore, improvements in programs through diverse audiovisual materials and self-study resources are necessary. Although scenario-based training and feedback devices positively impact CPR skill acquisition and performance [29,30], studies have found no relationship with patient outcomes [31,32]. Thus, further research is needed to develop and validate programs aimed at enhancing accurate CPR performance in middle-aged adults.

This study had some limitations. First, it was a secondary data analysis focusing solely on middle-aged adults in Korea, which limits the variability of variables and generalizability of the results. Second, the difference in sample sizes between the groups based on their CPR performance ability may have influenced the results, requiring cautious interpretation. The larger group size could have led to more statistically significant findings, potentially skewing the overall interpretation. Nevertheless, this study contributes to raising societal awareness and emphasizing the importance of the knowledge of MI early symptoms and CPR practical training experience by identifying factors affecting the CPR performance ability of middle-aged adults. This, in turn, can enhance emergency response capabilities and strengthen the overall safety net of society.

The literature clearly strengthens the association between the early recognition of MI symptoms and the success of life-saving efforts, such as CPR. Our findings elucidate previously unknown associations between the ability of middle-aged adults to recognize the early symptoms of MI, their CPR and AED training history, and their self-reported ability to complete CPR in emergencies successfully. Enhancing the recognition of early MI symptoms and providing hands-on CPR training is vital for improving CPR performance among middle-aged adults and enhancing the overall social safety net.

## 5. Conclusions

Recognizing the primary cause of cardiac arrest and MI, and performing accurate CPR promptly is essential to reduce mortality rates among patients undergoing cardiac arrest and increase the rate of return of spontaneous circulation. This study examined the correlation between MI early symptom recognition, CPR practical training experience, and CPR performance ability in 99,945 middle-aged adults aged 40–64 years. This study found that the increased recognition of early MI symptoms improves CPR performance, and practical training experiences with mannequins and AEDs strongly impact CPR performance. Therefore, developing and implementing programs that reflect individual characteristics is crucial to ensure that middle-aged adults can accurately perform CPR.

## Figures and Tables

**Table 1:** General characteristics of the participants (N = 99,945).

Variables	Total	CPR Proficiency Group (N = 15,477)	Comparison Group (N = 84,468)	χ^2^ or t(*p*)	Cramer’s V
	Mean (SD) or N (%)				
Sex					
Male	46,822 (46.8)	10,075 (65.1)	36,747 (43.5)	2449.15 (<0.001)	0.16
Female	53,123 (53.2)	5402 (34.9)	47,721 (56.5)		
Age (year)	53.11 (7.12)	52.98 (7.10)	53.14 (7.20)	2.51 (0.01)	
40s	33,382 (33.4)	5194 (33.6)	28,188 (33.4)		
50s	41,527 (41.6)	6684 (43.1)	34,843 (41.2)	35.526 (<0.001)	0.02
60s	25,036 (25.0)	3599 (23.3)	21,437 (25.4)		
Family composition					
1st generation	42,090 (42.1)	6464 (41.8)	35,626 (42.2)	1.06 (0.60)	0.003
2nd generation	52,523 (52.6)	8192 (52.9)	44,331 (52.5)		
3rd generation	5332 (5.3)	821 (5.3)	4511 (5.3)		
Educational level					
Lack of schooling	276 (0.3)	19 (0.1)	257 (0.3)	1036.49 (<0.001)	0.10
Elementary school graduate	4779 (4.8)	354 (2.3)	4425 (5.2)		
Middle school graduate	9630 (9.6)	941 (6.1)	8689 (10.3)		
High school graduate	41,810 (41.8)	5813 (37.5)	35,997 (42.6)		
College graduate or higher	43,450 (43.5)	8350 (54.0)	35,100 (41.6)		
Employment					
Employed	76,418 (76.5)	13,462 (87.0)	62,956 (74.5)	1126.16 (<0.001)	0.11
Unemployed	23,527 (23.5)	2015 (13.0)	21,512 (25.5)		
Place of residence					
City	29,259 (29.3)	3960 (25.6)	25,299 (30.0)	120.35 (<0.001)	0.04
Province	70,686 (70.7)	11,517 (74.4)	59,169 (70.0)		
CPR training experience					
None	41,191 (41.2)	800 (5.2)	40,391 (47.8)		
2 years ago	35,934 (36.0)	5446 (35.2)	30,488 (36.1)	159.86 (<0.001)	0.41
≤2 years ago	22,820 (22.8)	9231 (59.6)	13,589 (16.1)		

CPR, cardiopulmonary resuscitation.

**Table 2:** Recognition of the early symptoms of myocardial infarction (N = 99,945).

Variables	Total	CPR Proficiency Group (N = 15,477)	Comparison Group (N = 84,468)	χ^2^ or t(*p*)
	Mean (SD) or N (%)			
Total	3.82 (1.52)	4.11 (1.32)	3.81 (1.50)	−24.65 (<0.001)
Jaw, neck, and back pain				
Awareness	69,930 (70.0)	11,873 (76.7)	58,057 (68.7)	396.54 (<0.001)
Unawareness	30,015 (30.0)	3604 (23.3)	26,411 (31.3)	
Chest pain				
Awareness	91,020 (91.1)	14,386 (93.0)	76,634 (90.7)	79.65 (<0.001)
Unawareness	8925 (8.9)	1091 (7.0)	7834 (9.3)	
Arm and shoulder pain				
Awareness	60,853 (60.9)	10,713 (69.2)	50,140 (59.4)	533.88 (<0.001)
Unawareness	39,092 (39.1)	4764 (30.8)	34,328 (40.6)	
Dizziness				
Awareness	78,573 (78.6)	13,018 (84.1)	65,555 (77.6)	329.003 (<0.001)
Unawareness	21,372 (21.4)	2459 (15.9)	18,913 (22.4)	
Shortness of breath				
Awareness	85,391 (85.4)	13,692 (88.5)	71,699 (84.9)	135.03 (<0.001)
Unawareness	14,554 (14.6)	1785 (11.5)	12,769 (15.1)	

CPR, cardiopulmonary resuscitation.

**Table 3:** Correlation between the participants’ recognition of the early symptoms of myocardial infarction.

Variables	Jaw, Neck, and Back Pain	Chest Pain	Arm and Shoulder Pain	Dizziness	Shortness of Breath
Jaw, neck, and back pain	1				
Chest pain	0.38 (<0.001)	1			
Arm and shoulder pain	0.52 (<0.001)	0.30 (<0.001)	1		
Dizziness	0.46 (<0.001)	0.47 (<0.001)	0.44 (<0.001)	1	
Shortness of breath	0.41 (<0.001)	0.60 (<0.001)	0.37 (<0.001)	0.48 (<0.001)	1

**Table 4:** Factors influencing CPR proficiency using multinomial logistic regression.

Variables	Categories	Able to Perform Approximately			Able to Perform Accurately		
		Β	OR (95% CI)	*p*	Β	OR (95% CI)	*p*
Intercept		2.17		<0.001	1.82		<0.001
Recognition of early symptoms of myocardial infarction		0.09	1.09 (1.05–1.13)	<0.001	0.20	1.22 (1.18–1.26)	<0.001
CPR training experience							
Mannequin	Yes		1			1	
	No	−0.73	0.49 (0.43–0.56)	<0.001	−1.56	0.21 (0.18–0.25)	<0.001
AED	Yes		1			1	
	No	−0.62	0.54 (0.48–0.60)	<0.001	−1.47	0.23 (0.20–0.26)	<0.001
−2 Log likelihood = 340.46							
χ(*p*) = 2136.62 (<0.001)							
NagelKerke R^2^ = 0.11							

Reference = Unable to perform; CPR, cardiopulmonary resuscitation.

## Data Availability

The data are available in a publicly accessible repository that does not issue DOIs. Publicly available datasets were analyzed in this study. This data can be found here: https://chs.kdca.go.kr/chs/rdr/rdrInfoProcessMain.do (accessed on 24 December 2024).
